# Impact of teachers training on HIV/AIDS education program among secondary school students in Bangladesh: A cross-sectional survey

**DOI:** 10.1371/journal.pone.0181627

**Published:** 2017-07-24

**Authors:** Haribondhu Sarma, Mohammad Ashraful Islam, Jahidur Rahman Khan, Kamal Ibne Amin Chowdhury, Rukhsana Gazi

**Affiliations:** 1 Nutrition and Clinical Services Division, International Centre for Diarrheal Disease Research, Bangladesh (icddr,b), Mohakhali, Dhaka, Bangladesh; 2 National Centre for Epidemiology and Population Health, Research School of Population Health, The Australian National University, Acton, Australian Capital Territory, Australia; 3 Center for Bioinformatics Learning Advancement and Systematics Training (cBLAST), University of Dhaka, Dhaka, Bangladesh; 4 Health Systems and Population Studies Division, International Centre for Diarrheal Disease Research, Bangladesh (icddr,b), Mohakhali, Dhaka, Bangladesh; University of New South Wales, AUSTRALIA

## Abstract

**Background:**

In 2007, the Government of Bangladesh incorporated a chapter on HIV/AIDS into the national curriculum for an HIV-prevention program for school students. For the efficient dissemination of knowledge, an intervention was designed to train the teachers and equip them to educate on the topic of HIV/AIDS. The present study intended to understand the impact of this intervention by assessing the knowledge, attitudes and behaviours related to HIV/AIDS, among the targeted students.

**Methods:**

A cross-sectional survey was conducted with the students at randomly selected schools from two adjacent districts. Considering exposure to intervention, one district was assigned for intervention and the other as a control. In total, 1,381 students, aged 13–18 years (or above) were interviewed, 675 from the control areas and 706 from the intervention areas. Univariate and bivariate analyses were performed on the collected data.

**Results:**

A significantly higher proportion (p<0.001) of students in the intervention areas attended HIV/AIDS classes, demonstrated better knowledge and fewer misconceptions regarding the transmission and prevention of HIV. The same was derived regarding their attitude towards people living with HIV, as a higher proportion (p<0.001) responded positively, compared to the control groups of the study. Additionally, multinomial logistic regression analysis showed that students in intervention area were more likely to have good knowledge on HIV transmission (OR 2.71, 95% CI 1.74–4.22) and prevention (OR 2.15, 95% CI 1.41–3.26) compared to the students in the control areas.

**Conclusions:**

The training programme needs to be scaled up, since it is likely to have an impact among students; we have witnessed that the interventions particularly helped increase HIV/AIDS knowledge among students and positively change the students’ attitudes towards HIV/AIDS.

## Introduction

The vulnerability of young people to HIV/AIDS is a major public-health issue worldwide. While many factors contribute to the increase of its vulnerability, research has identified lack of knowledge as one of the leading issues [1–3]. A study conducted in Bangladesh reported that only 48% of male students were aware of at least two correct measures of preventing transmission of HIV, and the other 43% had knowledge regarding at least two correct modes of transmission [4]. Misconceptions about the spread of HIV from infected persons were found in over 40% of the students [5]. The Multiple Indictor Cluster Survey 2009 reported that the level of comprehensive and correct knowledge of HIV prevention decreased (15.8% in 2006 to 14.6%) among Bangladeshi women aged between 15–24 years [6], though the level of knowledge was found to be higher among urban women than those in the rural areas. To enrich the level of knowledge and reduce misconceptions among the students regarding HIV/AIDS, current prevention efforts are not confined to addressing only at-risk groups, but rather the goal is to reach the majority of the students before they make major life choices [7]. Here, the school-based programs offered the opportunity to encourage adolescents and young people to delay the onset of sexual activity and promote safe sexual behaviours via the usage of condoms, and increasing contraceptive usage after sexual initiation [8].

Findings from the evaluation studies on curriculum-based interventions across a wide range of nations, cultures, and student groups suggested that these programs could contribute to creating a significant impact on their knowledge and attitudes regarding sexual behaviours [9– 11]. Previous studies have also suggested that alteration of knowledge and attitudes through the curriculum-based HIV/AIDS education programme is an easier method compared to receiving positive impact through behaviour change methods [12]. Literature shows that sexual education and HIV-prevention programmes had positively changed the sexual behaviour of the students, for instance, it influenced the delay in initiation of sex and the increased usage of condoms [9, 13]. Study conducted in Bangladesh also found that students who had very good knowledge on HIV/AIDS, revealed textbooks, and teachers as their main sources of HIV/AIDS information [14].

**Fig 1:**
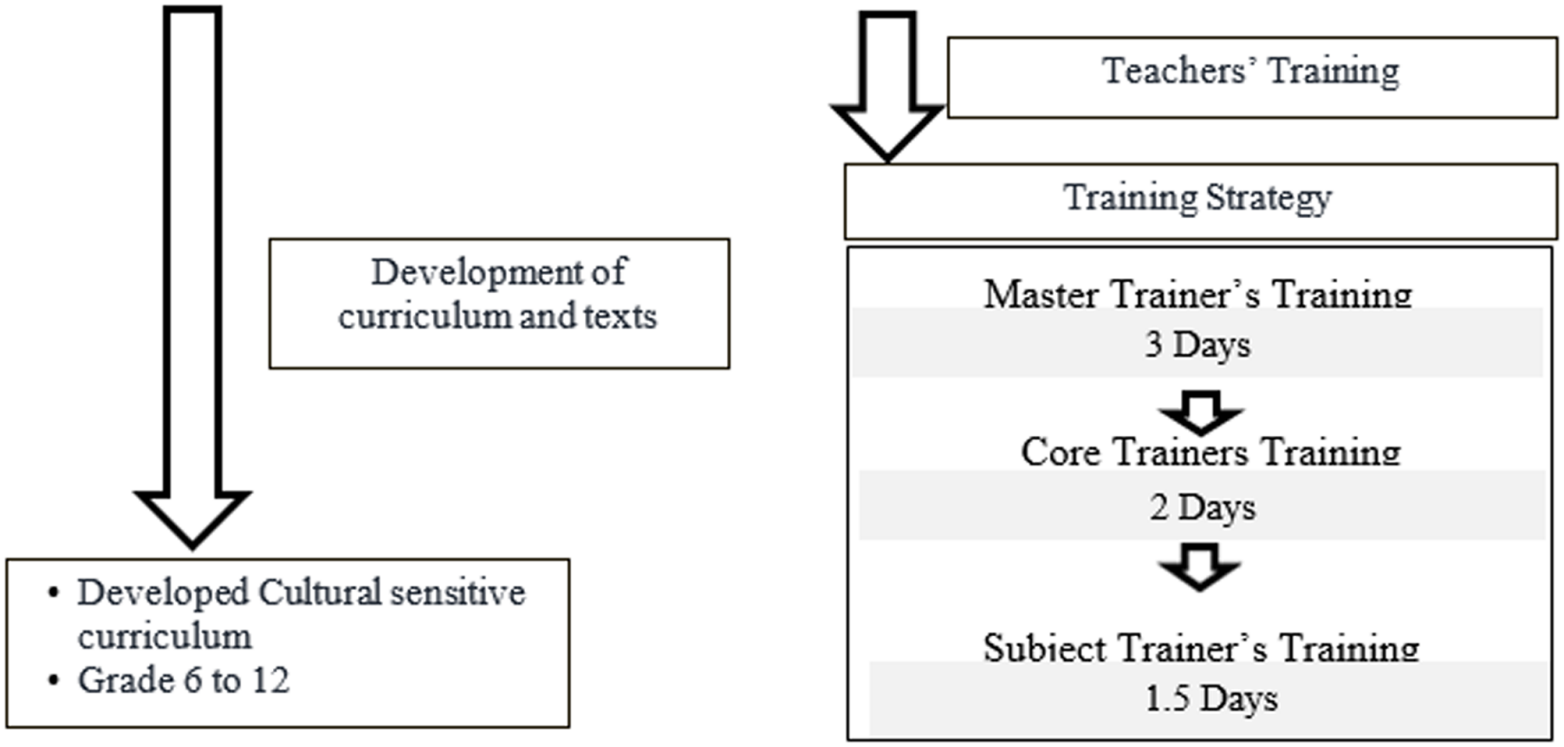
Cascaded training programme of PIACT Bangladesh.

PIACT Bangladesh, a non-profit, non-governmental organization (NGO) has implemented a programme, titled “Institutional capacity building through a nation-wide teachers training programme” from 2004. The above programme was commissioned under Government of Bangladesh’s (GoB) own project titled “Prevention of HIV and AIDS among young people in Bangladesh”. The GoB and partners developed an HIV/AIDS curriculum and the texts for grades VI-XII. Additionally, PIACT Bangladesh developed a cascaded training programme, and trained the teachers who would teach on the subject of HIV/AIDS at the classroom setting (Fig 1).

The broad objective of the training was to develop the skills and knowledge of teachers so that they could appropriately communicate the messages relating to HIV/AIDS in the classroom setting. The training sessions were focused on providing appropriate information on HIV/AIDS and on evolving ways of using interactive methods to teach the contents of the subject; for example, how to organise group discussions and puzzle games, and how to encourage active participation using role-play methods.

The impact this training has on the teachers abilities, skills and confidence to teach on HIV/AIDS in classroom has already been published [15, 16]. There are also published literatures on knowledge and attitudes of students of low and middle-income countries towards HIV/AIDS [17–22], and even a systematic review of school based sex education and HIV prevention in low and middle-income countries [23]. However, no study besides this has ever been conducted to assess the impact of the teachers’ training programme among the students, who are the final recipients of the prevention education. Therefore, the question was whether the teachers’ training on HIV/AIDS education would have any impact on knowledge, perception and attitudes related to HIV/AIDS among secondary-level school student in Bangladesh? Teachers’ training may have impact on the knowledge, attitudes and behaviours among the secondary level students in the low-HIV epidemic setting. Specifically, the objective of the study were to: a)to understand the participation of students in the HIV/AIDS class; b) to understand the knowledge and perceptions among students about HIV transmission and prevention; c) to understand attitudes, behaviours and believes among the targeted students relating to HIV/AIDS.

## Materials and methods

### Research design

**Fig 2:**
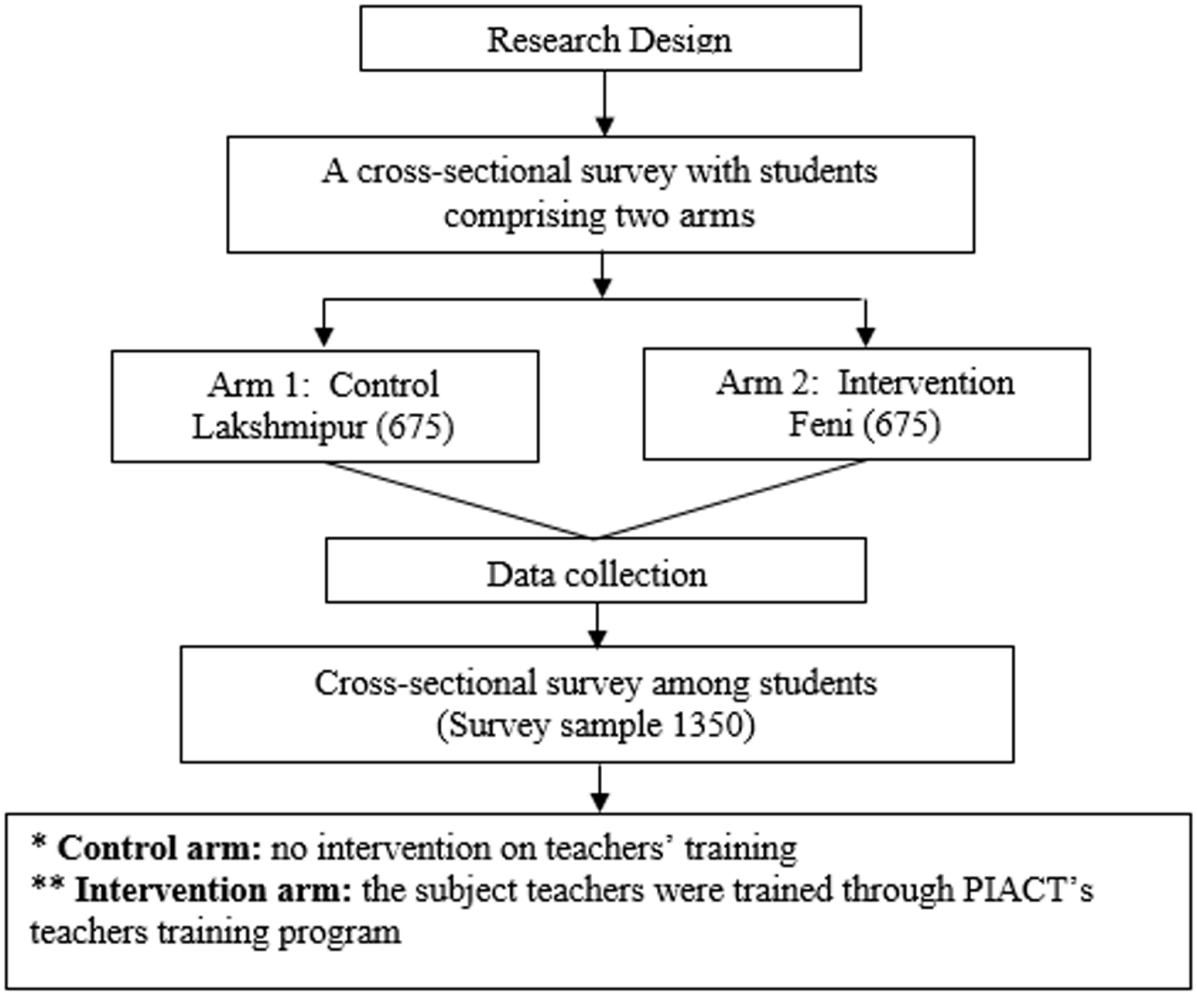
Research design.

This study was designed to be cross-sectional, comprising of two arms: a control arm and an intervention arm. In the ‘control’ arm, except for the availability of the HIV/AIDS curriculum, no intervention on the teachers training on HIV/AIDS was provided (Fig 2). Whereas, in the ‘intervention’ arm, in addition to providing the HIV/AIDS curriculum, PIACT Bangladesh provided training through the cascading teachers training programme on HIV/AIDS.

### Study sites

The study was conducted at two randomly selected districts of the Chittagong division. Lakshmipur was randomly drawn as the control area from the list of districts that were not being covered by cascading teachers training programme. Feni was selected as the intervention area from the list of districts, where the cascading training programme was conducted at least a year prior to the study.

**Fig 3:**
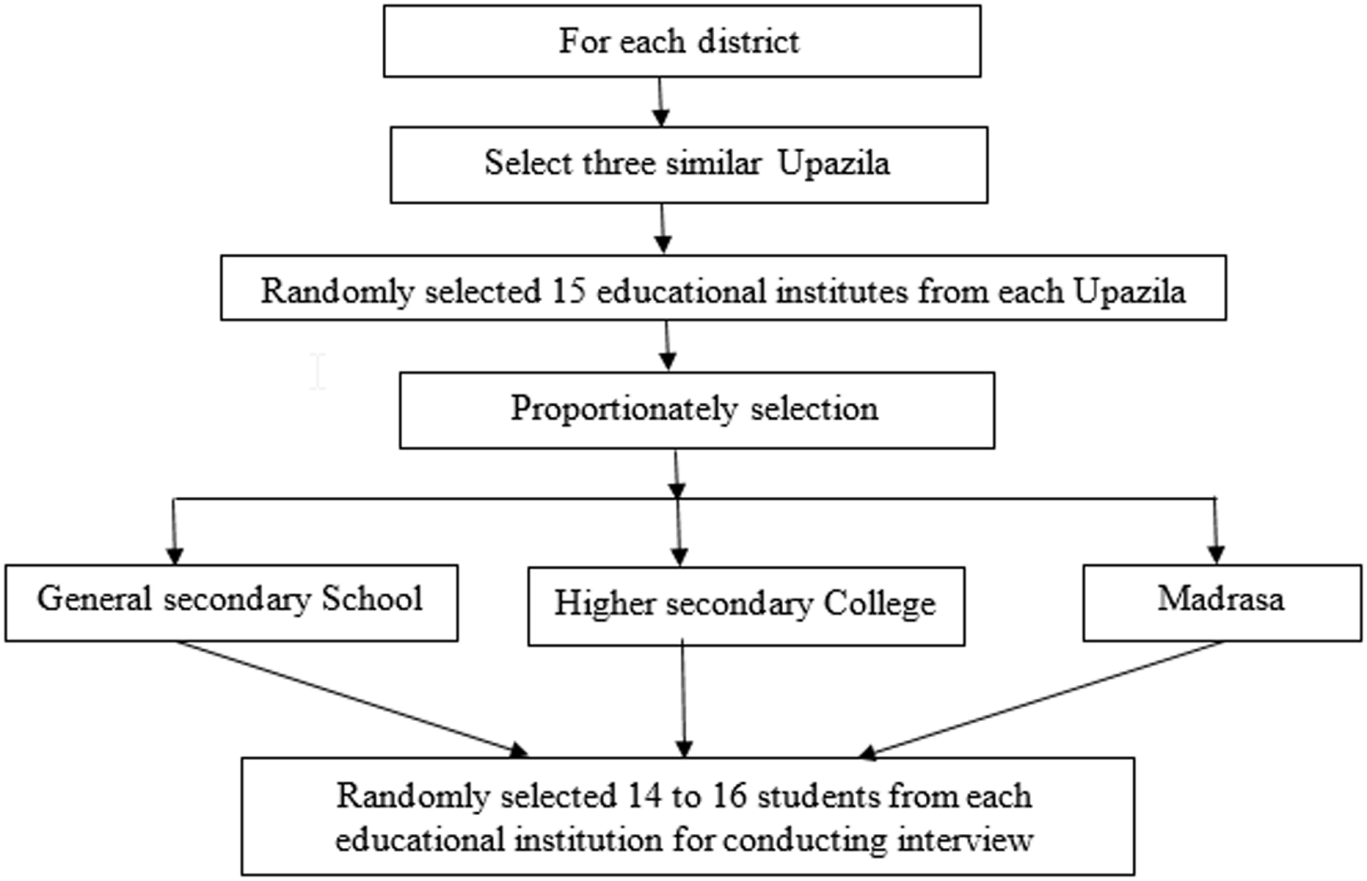
Recruitment process of the students.

From each district, three upazilas (sub-districts) were randomly selected. While selecting the upazilas, we considered selecting similar ones from each district, while excluding the sadar upazilas. We measured that the socio-cultural-economic variations were higher between the Sadar upazila and the other upazilas. We identified similar upazilas through the analysis of secondary data, such as the literacy rate, number of schools/colleges/*madrasha*s(Islamic shools) present, and the geographical distance from the districts’ headquarters. From similar short-listed upazilas, we randomly selected Raipur, Ramgati, and Ramganjupazilas, from the district of Lakshmipur, as the control sites, and Chagalnaiya, Fulgazi and Porshuramupazilas, from the district of Feni, as the intervention sites (Fig 3).The refusal case was low, a total of 6 children were refused in both the study areas.

### Inclusion criteria

- Student who studying in grade/year 8 to year 11
- Students who attended school during the day of interview

### Data-collection

Data for this study were collected from November 2010 to June 2011. The sample-size for the survey was calculated by taking the endline study conducted on students in the past into consideration, to learn the distribution of correct knowledge among studentscurrently in school [24]. We estimated the sample-size to be a sample of 1,350 students with 95% confidence level, 80% power, non-exposed: exposed ratio as 1:1, and 10% refusal cases. The sample between non-exposed (control) and exposed (intervention) students were distributed as 675 and 675, respectively. Within each district, the samples were equally distributed among the three randomly selected upazilas. The total sample from each upazila was proportionately distributed among schools, colleges, madrashas, and technical institutes. 15 educational institutes were then randomly selected from each upazila. From each selected institute, 14–16 students were randomly selected from Class IX and asked to participate in the survey. Half of them were male, and half were female. Six Field Research Assistants (FRA) and two Field Research Officers (FRO) from icddr,b were recruited and trained for collecting survey data, where the FROs supervised and monitored the overall data-collection activities in the field to ensure the quality of data.

### Ethical aspects

The Institutional Review Board of icddr,b, comprising two committees, they are named as: the Research Review Committee and the Ethical Review Committee, approved the study. The interviewers took written ascent from the students before conducting survey interviews. As the study was conducted in school setting, the research team also took written consent from the headmaster of the school as local guardian of students for conducting interview with his/her students.

### Survey instrument and measurement

To develop the questionnaire, we followed the CDC guidelines from The Handbook for Evaluating HIV Education[25]and adopted some items from other studies conducted among students in Bangladesh [24, 26–30], which addressed issues relating to HIV and sexually transmitted infections (STI).To be noted, the CDC guidelines were adopted whilst taking into consideration the socio-cultural context of Bangladesh. A 7-sections survey questionnaire was drafted, pre-tested and finalized; the sections included:-participation in HIV/AIDS class; knowledge, attitudes, beliefs and behaviours related to HIV/AIDS; and perception about peer exposure in risky behaviour.

The questionnaire contained a 25-item section to measure the functional knowledge on HIV/AIDS (i.e. knowledge necessary to reduce the risk of HIV infection). This instrument was employed in two ways. We used it for measuring the accuracy of students' knowledge on HIV/AIDS and for measuring the students' confidence in their knowledge on HIV/AIDS. To score this section for knowledge, we considered only one part (22-item) as to whether the participants indicate that an item is true or false or on whether they know or not if it is true or false.

For the students' beliefs, acceptance of, and attitudes towards, people who have AIDS, we used the 9-item section. The 25-item section measures the students' attitudes across five dimensions that are potentially related to whether a student might engage in HIV-risk behaviours. To measure the confidence of students in their ability to resist peer pressures, the 7-item section was used. The section attempts to assess refusal skills of the students within their age-appropriate social situations, and to make estimates about their friends' values and behaviours, the 15-item section was used. This section measures perceptions of students about their friends' behaviours and values relating to the possibility of being infected with HIV. This section was designed to measure HIV-risk behaviours of students. Responses to each of the 15 statements were interpreted separately. For the group of students assessed, the percentage of responses to the four choices for each item would determine, for example, the percentage of students who indicated that all their friends "have never had sex”; the percentage of students who indicated that most of their friends "have never had sex”; and so on. It is possible to collapse response categories, such as ‘All’ and ‘Most’ responses or ‘Some’ and ‘None’ responses. For measuring the students’ risk-behaviours relating to HIV/AIDS, we used the 8-item section based on two behaviour indicators: (a) the number of students who had exposure to sexual activity and (b) the number of students exposed to substance abuse, particularly drug abuse. This section is designed to measure the HIV-risk behaviours of students.

### Data analysis

The data were checked for consistency, and then compiled and the short-answer responses of the respondents were coded. The data was entered into the Epi Info software (version 3.3.2). Univariate and bivariate analyses were then performed using the R software (version 3.0.0).

We compared the key outcomes of the intervention, such as knowledge, attitudes, beliefs, and behaviours relating to HIV/AIDS of the student between the control and the intervention groups. Students from the control and the intervention areas reported different modes of HIV transmission and prevention. The responses regarding the modes of HIV transmission were categorized into the following five broad groups: (a) sexual mode of transmission; (b) receiving unscreened blood; (c) sharing needles/syringes for injecting drugs; (d) through breastfeeding by an HIV-infected mother; and (e) receiving HIV-infected organ/tissue for transplantation. Similarly, knowledge about modes of prevention was also categorized into the following four broad modes: (a) taking protection during sex; (b) avoiding HIV-infected/ unscreened blood transfusion; (c) using new syringes/ needles; and (d) stopping breastfeeding by HIV-positive mothers. Students got 1 point for correctly mentioning each of the modes of transmission and prevention. The total points obtained by a student represented the score for the knowledge they had regarding HIV transmission (T-score) and the score for the knowledge they had regarding HIV prevention (P-score). These scores were categorized as ‘poor’ (score <2), ‘moderate’ (score 2) and ‘good’ (score ≥3). Score categorization was done divided total score based on quintiles. We performed a chi-square test to find out the association between knowledge on modes of transmission as well as on prevention, with other covariates, such as household size, sex (girl, boy), reading news magazine and listening to the radio (no, <1 per week, frequently), attending class on HIV (yes, no), area (control, intervention), sources of information: parents/relatives, friend, radio/TV, textbook, teacher, and newspaper categorized as (yes, no). Then we fitted multinomial logistic regression with random effect to find out which risk factors correlate with knowledge on the mode of transmission and prevention. Random effect model is considered to adjust the cluster (school) level variation on outcome. We choose ‘poor’ knowledge as a reference category on both HIV transmission and prevention. Results from the regression analyses are presented as odds ratios (OR) with corresponding 95% confidence interval (CI). We also presented intra-cluster correlation coefficient (ICC) for presenting cluster level variability.

## Results

### Background characteristics of the students

In total, interviews with 1,381 students were conducted, 675 from the control areas and 706 from the intervention areas. A higher proportion of student in both the control (48%) and the intervention (51%) areas were aged 13–14 years; a similar pattern was seen in the 15–16 years age-group. The ratio of boys and girls in both the control and intervention areas was almost 50:50.Agriculture was the main occupation of 26% of the fathers of the respondents in both the study areas, followed by business (21%) and service (17%). In both the areas, more than 97% of the mothers of the students were housewives. Seventy-six percent of the students in both the areas read newspapers at least once a week; however, only 6% read the newspaper daily. The respondents who listened to the radio were more in number in the control areas (41%) than in the intervention areas (24%). A similar percentage of respondents watched TV in both the control (88%) and the intervention (92%) areas. However, there was no notable difference found from reading newspapers among the respondents (Table 1).

**Table 1:** Background characteristics of study participants, %.

Characteristics		Control			Intervention		
		Boys (n = 339)	Girls (n = 336)	Total (n = 675)	Boys (n = 349)	Girls (n = 357)	Total (n = 706)
**Age**	13–14 years	40	56	48	37	65	51
	15–16 years	53	43	48	54	34	44
	17–18 years or above	7	1	4	8	1	5
**Fathers’ occupation**	Agriculture	32	23	28	28	20	24
	Business	20	26	23	19	20	19
	Service	15	14	15	19	21	20
	Others	33	36	35	35	39	37
**Mothers’ occupation**	Housewife	98.5	95.5	97	96	96	96
	Others	1.5	4.5	3	3	4	4
**Access to mass media**	Read news paper	83	65	74	91	64	77
	Listening radio	47	35	41	24	24	24
	Watching TV	93	83	88	94	89	92

### Proportion of student attended in the HIV/AIDS class

About 70% of the students from both study areas attended in the HIV/AIDS classes. Proportionately, more students from the intervention areas (85%) attended in the HIV classes compared to the control areas (55%). More girls attended the HIV/AIDS classes in both the control (56%) and the intervention (90%) areas. The majority (58%) of the students from the areas stated that boys asked the teachers more questions while they are attended in the HIV/AIDS classes than the girls.

In addition, the types of questions asked by the students were very general, for instance, what do the abbreviations ‘HIV’ and ‘AIDS’ stand for (45%), why and how is HIV transmitted (63%), and the ways of preventing the spread of HIV/AIDS (31%). When the students were asked why they were not asking more questions in the class, 40% reported that they felt ‘ashamed’ and when asked about their feelings regarding learning about HIV/AIDS in a co-ed class, 67% of them reported that they felt ‘comfortable’ while, 32% stated that they felt ‘shy’.

### Students’ knowledge on HIV transmission and prevention

Almost every student from the intervention and control areas reported that they had heard about HIV/AIDS. When the respondents were asked about their knowledge on the modes of HIV transmission, sexual mode of transmission was mentioned by 79% and unscreened blood transmission was mentioned by 50% of them. The number of students who are aware about the transmission of HIV through sexual relationships was significantly higher in the intervention areas (p = 0.002) than in the control areas. Moreover, the students in the intervention areas were more knowledgeable about a couple of the other modes of transmission, such as unscreened blood (p<0.001), breastfeeding (p<0.001), and receiving HIV-infected organs (p<0.001), compared to the control areas. There were no differences found in the knowledge of students, on HIV prevention through protected sex, in any of the study areas. In regards to the other three modes of prevention, a significantly higher number of respondents (p<0.001) in the intervention areas reported correctly compared to the control areas.

When we asked about the sources of information on HIV/AIDS, 74% of the students mentioned textbooks, followed by class teachers (66%), and mass media such as radio/TV (65%). As a source of knowledge on HIV/AIDS, the class teacher was cited by a significantly higher proportion (p<0.001) in the intervention group compared to the control group (Table 2). The students also gathered knowledge about the transmission and prevention of HIV from textbooks, resulting in a significant impact (p = 0.03).

**Table 2:** Knowledge about HIV transmission and prevention, %.

Characteristics	Control group			Intervention group		
	Boys (n = 339)	Girls (n = 336)	Total (n = 675)	Boys (n = 349)	Girls (n = 357)	Total (n = 706)
Heard about HIV/AIDS						
**Coughing and sneezing spread HIV (false)**	99	96	97	100	100	100
	**Boys (n = 335)**	**Girls (n = 322)**	**Total (n = 657)**	**Boys (n = 349)**	**Girls (n = 356)**	**Total (n = 705)**
Knowledge about mode of transmission						
Sexual mode of transmission**	80	70	75	88	76	82
Receiving unscreened blood***	31	43	37	54	68	61
Sharing needles/syringes for injecting drugs**	28	29	29	44	26	35
Through breastfeeding by HIV- infected mothers***	19	22	20	44	44	44
Receiving HIV-infected organ/tissue for transplantation***	11	9	10	21	17	19
Knowledge about mode of prevention						
**Taking protection during sex**	69	50	60	68	52	60
Avoiding HIV- infected/ unscreened blood transfusion***	26	41	34	54	64	59
Using new syringes/needles***	48	46	47	65	63	64
Stopping breastfeeding by HIV/AIDS-infected mothers***	11	13	12	23	28	26
Source of HIV/AIDS knowledge						
Textbook*	72	70	71	87	65	76
**Radio/TV**	77	50	64	83	51	67
Class teacher***	50	49	49	80	83	82
**Friends**	30	16	23	24	16	20
Newspapers/magazines/leaflets*	35	11	23	45	14	29
Parents/relatives/others’ family members/neighbours*	13	22	17	9	17	13

*** p-value<0.001;

**p-value <0.01;

*p-value <0.05; p-values are based on total values in control and treatment groups.

According to the Table 3, the proportion of students with poor knowledge about HIV transmission among the students was about 37%, where 36% were good. In addition, the proportion of students with poor knowledge about HIV prevention was little higher than transmission, about 41%, where only 29% and 30% student had moderate and good knowledge about HIV prevention respectively.

**Table 3:** Distribution of knowledge of students regarding HIV transmission and prevention in different categories.

Knowledge about HIV transmission			
	**Poor (Score = 1)**	**Moderate (Score = 2)**	**Good (Score ≥ 3)**
Count	**513**	**370**	**498**
%	**37.15**	**26.79**	**36.06**
Knowledge about HIV prevention			
	**Poor (Score = 1)**	**Moderate (Score = 2)**	**Good (Score ≥ 3)**
Count	**563**	**407**	**411**
%	**40.77**	**29.47**	**29.76**

Findings from regression analysis showed that the number of factors that were significantly associated with knowledge of students regarding HIV transmission and prevention. Students from intervention areas were 2.71 times more likely to have better knowledge regarding HIV transmission than students from the control areas. Female students were more likely to have better knowledge about HIV transmission than their male counterpart (OR1.88, 95% CI 1.31–2.68). Attendance in HIV/AIDS classes was highly associated with the students having good knowledge (OR 3.92, 95%CI: 2.16–7.11). Textbooks were a great source of acquiring good knowledge regarding HIV transmission, which means students from both intervention and control areas were benefited from text- books as available through the HIV/AIDS curriculum in both areas. However, teachers being cited as a significant source of knowledge on HIV transmission mean that students in the intervention areas were further benefited from those teachers who were the primary subject of this intervention. Regarding knowledge about HIV prevention almost similar trends were observed among the factors. Results suggest that a significant effect of cluster or school on outcome variable. The variances of the random coefficients are statistically significant and suggesting substantial variation between schools in students' knowledge of HIV/AIDS transmission and prevention. For example, intra-cluster correlation (ICC) 0.159 suggests that 15.9% of the variability in knowledge about HIV transmission lies between schools, where ICC 0.115 suggests that 11.5% of the variability in knowledge about HIV prevention lies between schools (Table 4).

**Table 4:** Multinomial logistic regression with random effect for knowledge about HIV transmission and prevention.

Variables	Knowledge about HIV transmission						Knowledge about HIV prevention					
	Moderate (Score = 2)			Good (Score ≥ 3)			Moderate (Score = 2)			Good (Score ≥ 3)		
	OR	95% CI	p value	OR	95% CI	p value	OR	95% CI	p value	OR	95% CI	p value
Intervention district **(Feni)**	1.94	1.24–3.02	**	2.71	1.74–4.22	***	1.66	1.11–2.50	*	2.15	1.41–3.26	***
House hold size	0.92	0.86–0.99	*	0.93	0.87–0.99	*	0.92	0.86–0.98	*	0.92	0.86–0.98	*
Sex: ***girl***	1.60	1.13–2.27	**	1.88	1.31–2.68	**	-	-	-	-	-	-
Parents/and relatives: ***yes***	-	-	-				1.72	1.15–2.58	**	1.46	0.94–2.27	0.096
Friend: ***yes***	1.02	0.69–1.51	0.906	1.68	1.15–2.46	**	1.29	0.89–1.87	0.172	1.71	1.17–2.50	**
Radio TV: ***yes***	2.10	1.49–2.95	***	3.22	2.26–4.6	***	2.18	1.58–3.00	***	2.25	1.60–3.16	***
Textbook: ***yes***	3.17	1.72–5.84	***	6.05	3.38–10.83	***	2.67	1.55–4.61	***	4.48	2.63–7.63	***
Teacher: ***yes***	1.18	0.7–1.96	0.535	1.69	0.99–2.88	0.055	1.33	0.81–2.18	0.264	1.60	0.94–2.73	0.080
Newspaper: ***yes***	0.93	0.62–1.39	0.711	1.63	1.11–2.4	*	1.03	0.71–1.48	0.892	1.58	1.08–2.29	*
News magazine:												
***Frequently***	0.79	0.53–1.17	0.239	1.42	0.93–2.17	0.107	1.44	0.98–2.13	0.067	1.52	1.00–2.31	*
***<1 per week***	1.21	0.80–1.84	0.359	1.85	1.18–2.89	**	1.81	1.21–2.69	**	1.66	1.08–2.55	*
Listen Radio:												
***Frequently***	-	-	-	-	-	-	0.44	0.25–0.79	**	0.89	0.53–1.51	0.677
***<1 per week***	-	-	-	-	-	-	0.99	0.69–1.40	0.939	1.14	0.78–1.65	0.501
Attend class on HIV: ***yes***	1.81	1.05–3.13	*	3.92	2.16–7.11	***	2.11	1.24–3.58	**	4.55	2.50–8.26	***
Variance of random effect	0.620						0.429					
Intra-cluster correlation (ICC)	0.159						0.115					

*** p-value <0.001;

**p-value <0.01;

*p-value <0.05

### Misconceptions related to HIV transmission and prevention

Common misconceptions relating to the transmission and prevention of HIV still remain among the students of both control and intervention areas. A significantly higher number of correct responses on common misconceptions were found from the intervention areas compared to the control areas. For example, in regards to the spread of HIV through coughing and sneezing, 47% of the students in the control areas correctly responded, however, a significantly higher (p<0.001) number of students in the intervention are as responded correctly (Table 5).

**Table 5:** Common misconception related to HIV transmission and prevention %.

Characteristics	Control group			Intervention group		
	Boys (n = 335)	Girls (n = 322)	Total (n = 657)	Boys (n = 349)	Girls (n = 356)	Total (n = 705)
**Correct response on common misconceptions**						
Coughing and sneezing spread HIV (*false*)***	45	49	47	71	73	72
**Sharing food/water with someone who has HIV (*false*)** ***	58	64	61	82	92	87
**There is a vaccine that can prevent from getting HIV (*false*)** ***	31	39	35	59	61	60
**Taking antibiotics can prevent HIV (*false*)** ***	38	48	43	51	68	60
**Taking bath in the same pond with a person who has HIV (*false*)** ***	68	76	72	88	92	90
**AIDS is a curable disease (*false*)** ***	61	66	63	74	84	79
**Washing genitals/ private parts, keeps a person from getting HIV (*false*)** ***	43	43	43	57	57	57
**HIV can be transmitted through touching/care giving PLHIV (*false*)** ***	68	73	70	87	91	89

*** p-value<0.001;

**p-value <0.01;

*p-value <0.05; p-values are based on total values in control and treatment groups.

Similarly, only half of the students from both groups could give correct responses on the two most common misconceptions—‘taking antibiotics can prevent HIV’ (51%) and ‘washing genitals/private parts can keep a person from contracting HIV’ (50%) (Table 5).

### Attitudes towards and beliefs relating to HIV/AIDS

The students from both the study areas showed positive attitudes towards PLHIV(people living with HIV) and positive beliefs relating to the transmission and prevention of HIV. However, on most of the attitude-related items, the proportion of students who reported positively was higher in the intervention areas compared to in the control areas (in all items p<0.001). About 70% of the students in the control areas reported that they would not feel risky being in the same classroom with someone who was infected with HIV. A significantly (p<0.001) higher proportion (92%) of the students from the intervention areas also reported the same. About 63% of the students from the control areas and 88% from the intervention areas felt that a person who has been infected with HIV should be allowed to eat with everyone else in the school cafeteria. More than 60% of the students in the control areas and 81% of the students in the intervention areas felt that they would not feel at risk when hugging a close friend who has been infected with HIV. Similarly, positive attitudes of the students towards PLHIV were also observed in different settings; PLHIV should be allowed to play sports in public places and even in the classroom (Table 6).

**Table 6:** Positive attitudes, beliefs and perceived risk of students about HIV/AIDS/STI transmission and prevention, %.

Characteristics	Control group			Intervention group		
	Boys (n = 335)	Girls (n = 322)	Total (n = 657)	Boys (n = 349)	Girls (n = 356)	Total (n = 705)
**Positive attitudes towards HIV**						
**I would not feel risk being in the same classroom with someone who has HIV. (*agree*)** ***	67	72	70	88	95	92
**A person who has HIV should not be allowed to eat with all in the school cafeteria. (*disagree*)** ***	60	66	63	85	90	88
**I would not feel risk in hugging a close friend who has HIV. (*agree*)** ***	57	63	60	72	89	81
**I would not feel risk in playing sports with someone who has HIV. (*agree*)** ***	73	72	72	86	94	90
**A person who has HIV should stay away from public places. (*disagree*)** ***	51	69	60	76	88	82
**I would usually avoid a classmate who I heard had HIV. (*disagree*)** ***	64	79	72	87	94	90
**I would avoid a classmate whose family member had HIV. (*disagree*)** ***	70	84	77	89	96	92
**Positive beliefs related to HIV/AIDS**						
**It is okay not to have sex while you are a teenager. (*agree*)** ***	91	87	89	95	97	96
**A teenager can inject drugs occasionally without a risk of being infected with HIV. (*disagree*)** **	64	60	62	60	81	70
**Teenagers should learn how to resist pressures of premarital sex from their friends. (*agree*)** **	86	95	91	91	99	95
**Teenagers should realize that if they are not careful about HIV, they could be infected with HIV. (*agree*)** *	90	98	94	94	99	97
**Anyone who shares needles during injecting drug has a chance of being infected with HIV. (*agree*)** ***	90	85	87	93	94	93
**HIV/AIDS class makes a lot of sense to wait to have sex until you get married. (a*gree*)** ***	50	53	51	71	88	79
	Boys (n = 339)	Girls (n = 336)	Total (n = 675)	Boys (n = 349)	Girls (n = 357)	Total (n = 706)
**Perceived risk about STI/HIV infection**						
**It is likely that I could at some time in the future get an STI infection from sex. (likely)** **	14	9	11	4	9	7
**It is likely that I could at some time in the future get HIV. (likely)** *	5	10	8	2	8	5
**I am very healthy so my body can fight off an HIV infection. (disagree)**	81	69	75	72	84	78
**I am not worried that I might get an HIV infection. (disagree)**	56	42	49	54	54	54
**Perception about peers**						
Peers’ habits in reading pornography***	58	46	52	51	30	41
Peers’ perception on sex before marriage**	19	10	14	8	10	9
**Ever sexual exposure of peer**	18	20	19	13	17	15

*** p-value<0.001;

**p-value <0.01;

*p-value <0.05; p-values are based on total values in control and treatment groups.

Similar to our findings on attitudes, the students in both the study arms had positive beliefs relating to the transmission and prevention of HIV. However, in all belief-related items, the proportion of students who reported positively was significantly higher (p<0.001) in the intervention areas compared to in the control areas. For example, about 89% of students in the control areas and 96% in the intervention areas believed in not having sex whilst they were teenagers (Table 6).

## Discussion

The positive impact of a HIV/AIDS education programme is not a new issue–especially in a country with a high prevalence of HIV. There were many studies, conducted in a setting with a generalized HIV epidemic, which suggested that the curriculum based HIV/AIDS education program would have a significant impact on improving knowledge, increasing positive attitudes, and reducing risky sexual behaviours related to HIV transmission and prevention, among the students [9–13]. However, this is not known for certain in the countries with low prevalence of HIV. In this context, this study has added new evidence. Despite the low HIV-prevalence status, the comprehensive HIV/AIDS curriculum with training for teachers on the curriculum had significant impacts among the students. The findings of this study showed that the intervention programme had significantly improved knowledge on the transmission and prevention of HIV, resulting in fewer misconceptions, and students in the intervention areas developed more tolerant attitudes towards HIV and AIDS compared to the students in the control areas. However, curriculum alone might have had an effect among students, as most students in both the study arms, were found to be aware of sexual behaviours and about preventing HIV from sexual transmission. It was important to know as a separate study conducted for baseline assessment of HIV/AIDS prevention programme in Bangladesh, pre-marital sex was reported to be substantially higher among the students [24].

Studies found that primary sources of information for students about HIV/AIDS were the print and electronic media [14–23, 31], whereas textbooks, and teachers were the next important sources of information [20, 23]. In this current study however, no notable differences were found among students from both the control and intervention areas, in regards to exposure to TV/radio/newspaper. Therefore, it can be concluded that, instead of other external sources, the teachers training had a good impact in increasing knowledge among students of intervention areas, as they stated teachers as one of the main sources of information. The above finding is strongly associated with the recent systematic review that showed students who received school-based sex education interventions had significantly greater HIV knowledge, condom use, and fewer sexual partners [23].

Successful implementation of the curriculum-based HIV education programme is not an easy task, even in the settings where HIV is generalized as an epidemic. The findings of this study revealed that in terms of participation of the students in the HIV/AIDS classes, proportionately more students from the intervention areas participated in the HIV classes compared to the students from the control areas. This means that this intervention might have an impact on increasing the skills of the teachers in ensuring the participation of students in the HIV classes. Previous studies have suggested that teachers need to be skilled in imparting sensitive information, such as HIV transmission and prevention, to their students [15, 16, 32]. The majority of students of both the arms felt ‘comfort’ attending co-ed classes, although more than half felt ‘ashamed’ to ask questions; the reason behind this could be that the girls are not accustomed to being outspoken in a wider audiences [33].

Regression analysis showed that the knowledge level of students about modes of transmission and prevention significantly improved in the intervention areas compared to in the control areas. In addition, the level of the students’ HIV/AIDS knowledge regarding modes of transmission and prevention were significantly associated with the students’ gender, the sources of information (friends, radio-TV, textbook, paper, teachers, magazine), household size, and participation in HIV/AIDS classes. These findings also correlate with other studies [34–37]. The results of this study also indicating that girls were more likely to have good knowledge than boys, while more girls were attended in the HIV/AIDS class compared to the boys. However, Multiple Indicator Cluster Survey 2009 indicating that knowledge of HIV prevention was low among women 15–24 years [38].

On the other hand, having good knowledge on HIV/AIDS could have other benefits in the development of adolescent as a whole. Since study participants came from young adolescent to older adolescent they might get benefits from the HIV/AIDS education. Previous literature on sexual education suggested that it helps to prevent and reduce the risks of adolescent pregnancy, HIV, and sexually transmitted infections for children and adolescents [39].

## Limitations

This is a cross-sectional survey and we do not have any baseline measure. Thus, there is no way of certainly knowing that the observed impact effects do result from the intervention or from any external factors. Another limitation of this study is that, the focus is only on knowledge, attitudes and perceptions–not on actual behaviours related to HIV prevention or more broadly, on reproductive health. In addition, as the students’ ages range from 13–18 years (or above), there is a possibility of bias in outcomes because of cognitive/comprehension level differences in understanding the HIV/AIDS knowledge, attitudes, and behaviours among older and junior students. However during enrolment, we excluded those who did not pass class IX in the previous year in order to avoid possible bias through longer exposure to the HIV/AIDS curriculum. Data are only representative of young students who attend schools in the surveyed sub-districts. The data are not generalizable across Bangladesh or representative of all secondary school students in the age groups. To better understand the outcomes and impact of the intervention, a pre-post assessment is needed using nationally-representative samples.

## Conclusions

Overall, the interventions created a positive impact among the students from the intervention areas, who are the final recipients of information about HIV/AIDS. It is likely that the intervention particularly helped increase the participation of the students during the HIV/AIDS classes, enhance their knowledge on HIV/AIDS, reduce their misconceptions about HIV/AIDS, and positively change their attitudes towards PLHIV.

The issue of self-risk perception relating to HIV infection implicates the need for further consideration during the future revision of the curriculum. However, all things considered, the cascading training programme needs to be scaled up, as it is more likely to have an impact among students, as the interventions particularly helped increase the knowledge of students and changed their attitudes positively towards HIV/AIDS.In order to have a better understanding of the outcome and impact of this intervention on students’ behavior, a pre-post assessment is needed to be done by using the nationally representative samples.
